# The Fleming Fund: Key Features of Design and Approach to Strengthening Antimicrobial Resistance Surveillance in Low- and Middle-Income Countries

**DOI:** 10.1093/cid/ciag499

**Published:** 2026-10-19

**Authors:** Tim Shorten, Jemima Clarke, Archibald Drake, Ankur Gupta-Wright, Milena von und zur Mühlen, Evelyn Wesangula

**Affiliations:** Itad Ltd, Brighton, United Kingdom; Independent Consultant, Redgrave, United Kingdom; Department of Health and Social Care, London, United Kingdom; Itad Ltd, Brighton, United Kingdom; Independent Consultant, Dar es Salaam, Tanzania; Itad Ltd, Brighton, United Kingdom; Independent Consultant, Bristol, United Kingdom; Department of Infectious Disease, Imperial College, London, United Kingdom; Department of Infectious Diseases and Tropical Medicine, University of Heidelberg, Heidelberg, Germany; Department of Infectious Diseases, Bristol NHS Foundation Trust, Bristol, United Kingdom; Department of Health and Social Care, Leeds, United Kingdom; Itad Ltd, Brighton, United Kingdom; Independent Consultant, Arusha, Tanzania; East Central and Southern Africa Health Community, Arusha, Tanzania

**Keywords:** Antimicrobial resistance, AMR surveillance, health systems strengthening, one health, evidence to action, LMIC, Fleming Fund, global health security

## Abstract

This paper examines the Fleming Fund as a major long-term investment in strengthening antimicrobial resistance (AMR) surveillance in low- and middle-income countries. Established by the UK Department of Health and Social Care in 2015, the Fund committed up to £498 million to support surveillance capacity across 25 countries in Africa and Asia through a One Health approach spanning human, animal and environmental health. The paper situates the Fleming Fund within wider global concern about AMR and highlights surveillance as essential for stewardship, policy and international coordination. It describes the Fleming Fund's design logic, governance arrangements, grant architecture and evolving strategic priorities, with particular attention to how the program combined laboratory strengthening, workforce development, data systems, quality assurance, sampling pathways and multisectoral coordination to generate credible and usable AMR data. The analysis also examines the Phase 2 shift from an initial emphasis on data generation toward greater attention to data interpretation, dissemination and use in policy and practice. Drawing on program documentation, evaluation evidence and stakeholder perspectives, the paper presents the Fleming Fund as an important case of large-scale surveillance system strengthening in resource-constrained settings. It argues that the Fund offers wider lessons on the time, coordination and institutional embedding required to move from externally supported capacity building toward sustainable national surveillance functions. These lessons are relevant to future AMR investments and to broader debates on global health security, health systems strengthening and One Health implementation.

Antimicrobial resistance (AMR) is a critical global health challenge that threatens progress toward universal health coverage, health security, and the Sustainable Development Goals. Since the 2016 O’Neill report “*Tackling Drug-Resistant Infections Globally*” [1], this assessment has gained traction internationally, including with the World Health Organization (WHO) and United Nations.

A consistent theme in global AMR policy has been the centrality of surveillance: without routine, comparable data on resistance patterns and antimicrobial use, governments and partners lack the evidence needed to prioritize interventions, target stewardship, and monitor progress [1]. The O’Neill report also underlined the need for a multisector or One Health response [1] implying surveillance capacity across human health, animal health and environmental sectors.

Strengthening AMR surveillance systems is technically complex and resource-intensive. It requires sustained investment in laboratory infrastructure, a skilled workforce, governance and coordination, and interoperable data systems. In many low- and middle-income countries (LMICs), where the burden of AMR is highest [2, 3], these foundational elements have historically been under-resourced or supported through fragmented, time-bound external initiatives, limiting continuity, standardization, and country ownership.

It is in this context that the Fleming Fund was established: as one of the largest long-term investments in AMR surveillance in LMICs, and large-scale effort to build national One Health surveillance capabilities at scale. Implemented across diverse national and regional contexts in Africa and Asia, the Fleming Fund sought to address long-standing constraints in laboratory microbiology capacity, data generation, workforce development and coordination across the human, animal and environmental health sectors, while linking country systems to regional and global surveillance systems.

The Fleming Fund was conceived as a long-term commitment, established in 2015 and implemented between 2017 and 2025 (with some activities continuing into early 2026), with a commitment up to £498 million. Its scale and duration (supporting work in 25 countries) make it a significant case for examining what it takes to build microbiology and AMR surveillance functions, integrate One Health considerations, and move from capacity building toward routine generation and use of surveillance data.

## ABOUT THIS SERIES

This series brings together evidence and learning from one of the largest sustained investments in AMR surveillance in LMICs. It aims to contribute to wider learning on how complex global health programs translate technical capacity into institutional and policy change, and to strengthen the use of evaluative approaches within UK-funded AMR programming and the wider literature.

The papers in this journal series were commissioned and funded by DHSC, delivered through a drafting group process convened by Itad (the Fleming Fund independent evaluation supplier) to collaborate on the content for each paper. The purpose is to synthesize learning from the program implementation and evaluation to inform future AMR surveillance and action, further strengthening the Fleming Fund's legacy. Rather than treating the Fleming Fund as a program assessed in isolation, the series uses it as a lens on broader questions. These include how AMR surveillance systems are built, strengthened, and used in resource-constrained settings.

This paper provides an overview of key features of the Fleming Fund's design and operations. There are five other papers in the series, including:

- Data generation—how the Fleming Fund combined laboratory, workforce, quality assurance, and data-system investments to increase the quantity and credibility of AMR surveillance data [4].
- Data use—how countries and partners have interpreted, communicated, and applied AMR evidence (and what still constrains routine decision-making use) [5].
- One Health—what multisectoral surveillance strengthening has meant in practice across human, animal and environmental domains [6].
- Kenya—a country case study tracing how program choices played out through national institutions, laboratories, and governance arrangements [7].
- The future of AMR surveillance—drawing together lessons from the program to suggest how surveillance systems can transition from externally supported capacity building to sustainable, nationally owned public health functions linked to action on AMR [8].

The series draws on this experience to examine how surveillance capacities are developed, how data and information flows can be established, and the conditions under which surveillance outputs are interpreted and used in decision-making. Particular attention is given to the interface between technical capability and governance, recognizing that the production of surveillance data alone is insufficient to ensure its routine or effective use. In this respect, the papers place greater emphasis on institutional, governance and political economy dimensions of AMR surveillance than is typical in a literature often focused primarily on technical system components.

The analysis is based on a structured narrative synthesis of program documentation, evaluation evidence and stakeholder perspectives. This approach enables cross-cutting analysis across diverse country contexts and components of the Fleming Fund, but it is subject to recognized limitations, including reliance on available program documentation and constrained ability to establish causal attribution.

## METHODOLOGY

### Approach

This series presents a structured synthesis of learning from the implementation of AMR surveillance strengthening activities supported through the Fleming Fund. The selection of papers is structured to maximize relevance to the intended audience, framed by the Fleming Fund's theory of change (ToC) and covering complementary components of surveillance system development and data use.

### Evidence Base

The analysis draws on program documentation, independent evaluation outputs and selected academic and grey literature, including surveillance reporting to international platforms. Evidence was selected purposively rather than comprehensively. Sources were prioritized where they provided insight into system functionality, implementation experience and reported outputs.

### Authorship and Drafting Process

Writing groups were convened through a structured invitation process designed to bring together contributors with extensive implementation experience from across the Fleming Fund. This process was led by a lead or coordinating author from the Itad evaluation team (Itad, www.itad.com, was the independent evaluation and learning partner for the Fleming Fund from 2016 to 2026; DHSC asked Itad to coordinate and facilitate the production of this series as an extension to this role), with support from an Itad “secretariat” function.

Contributors included country-based implementers, policy-makers, laboratory specialists, clinicians and program partners across human, animal and environmental health sectors, alongside regional and global experts. Collectively, contributors brought experience from the design, implementation and evaluation of AMR surveillance systems across multiple countries, sectors and stages of system development. Drafts were developed through successive rounds of discussion, review and revision, allowing contributors to challenge interpretations, refine arguments, and agree conclusions.

### Scope and Limitations

The series is intended as a practice-informed synthesis rather than an independent evaluation. It does not seek to establish causal attribution or provide a comprehensive account of program performance; rather, it uses available evidence to identify lessons and insights relevant to the AMR community. This entails inherent limitations, including potential biases associated with program-linked authorship, purposive evidence selection, and reliance on documented and reported experience. Where available, evidence on constraints, partial implementation, and areas of limited progress was considered and presented, although decisions about inclusion and emphasis were ultimately made through discussion and consensus within the drafting groups. The drafting groups were mostly led by UK-based authors, but group membership was intentionally broader, ensuring that synthesis and interpretation were grounded in contributions from country-level implementers and decision-makers. These considerations were recognized in the design of the series and are presented transparently, alongside explicit statements of conflict of interest.

## PURPOSE AND OBJECTIVES OF THE FLEMING FUND

The Fleming Fund was established in 2015 by the UK Department of Health and Social Care (DHSC) as a multiyear investment to strengthen the generation, analysis, sharing and use of AMR surveillance data in LMICs.

The Fleming Fund's objectives can be summarized as follows:

- to strengthen laboratory capacity and surveillance systems for AMR, antimicrobial consumption, and antimicrobial use across One Health sectors;
- to support countries to generate high-quality, standardized AMR data aligned with international reporting systems, including WHO's GLASS;
- to improve the analysis, interpretation, sharing, and use of AMR data to inform policy, clinical practice, and investment decisions;
- to contribute to sustainable national surveillance systems that are embedded within broader health systems, governance arrangements, and national AMR action plans.

The Fleming Fund was designed to generate benefits at national, global, and UK levels simultaneously. For partner countries, investments focus on strengthening laboratory capacity, surveillance systems, and the routine generation and use of AMR data to inform policy, clinical practice, and stewardship. At the global level, these investments contribute to a more complete and comparable evidence base on AMR, supporting international reporting systems and collective action. For the United Kingdom, the rationale is rooted in the transboundary nature of antimicrobial resistance: improving the scope, quality and use of surveillance data in LMICs strengthens the global evidence base on which the UK depends to understand emerging resistance patterns, assess risks, and inform its own public health and policy responses. Taken together, the Fleming Fund reflects an approach in which national capacity strengthening and global health security are closely aligned, rather than separate objectives.

These objectives were pursued through a One Health approach, recognizing the interconnected nature of human, animal, and environmental drivers of AMR and the need for coordinated governance across sectors. One Health formed an underpinning principle for the Fleming Fund, alongside country ownership, alignment, sustainability, gender, and equity.

## STRUCTURE AND GOVERNANCE OF THE FLEMING FUND

### Governance

The Fleming Fund was funded and overseen by DHSC, through a London-based team, with leadership from the UK's Special Envoy on AMR, Dame Sally Davies. The DHSC team leveraged existing diplomatic, technical and partner networks at country level; a program board in DHSC supported on management and accountability, and a Technical Advisory Group [9] guided the Fleming Fund's approach.

The majority of Fleming Fund investments (c.70%; see Figure 1) were managed by Mott Macdonald as the Fleming Fund's Management Agent (MA) through a core team in London and regional hubs in Accra, Kampala, New Delhi and Bangkok. The MA was responsible for designing, procuring, and managing a portfolio of country and regional grants alongside a Fellowship scheme (described in more detail in the following section). The DHSC team also managed a set of direct grants, described below, to complement the MA-managed investments.

**Figure 1.**
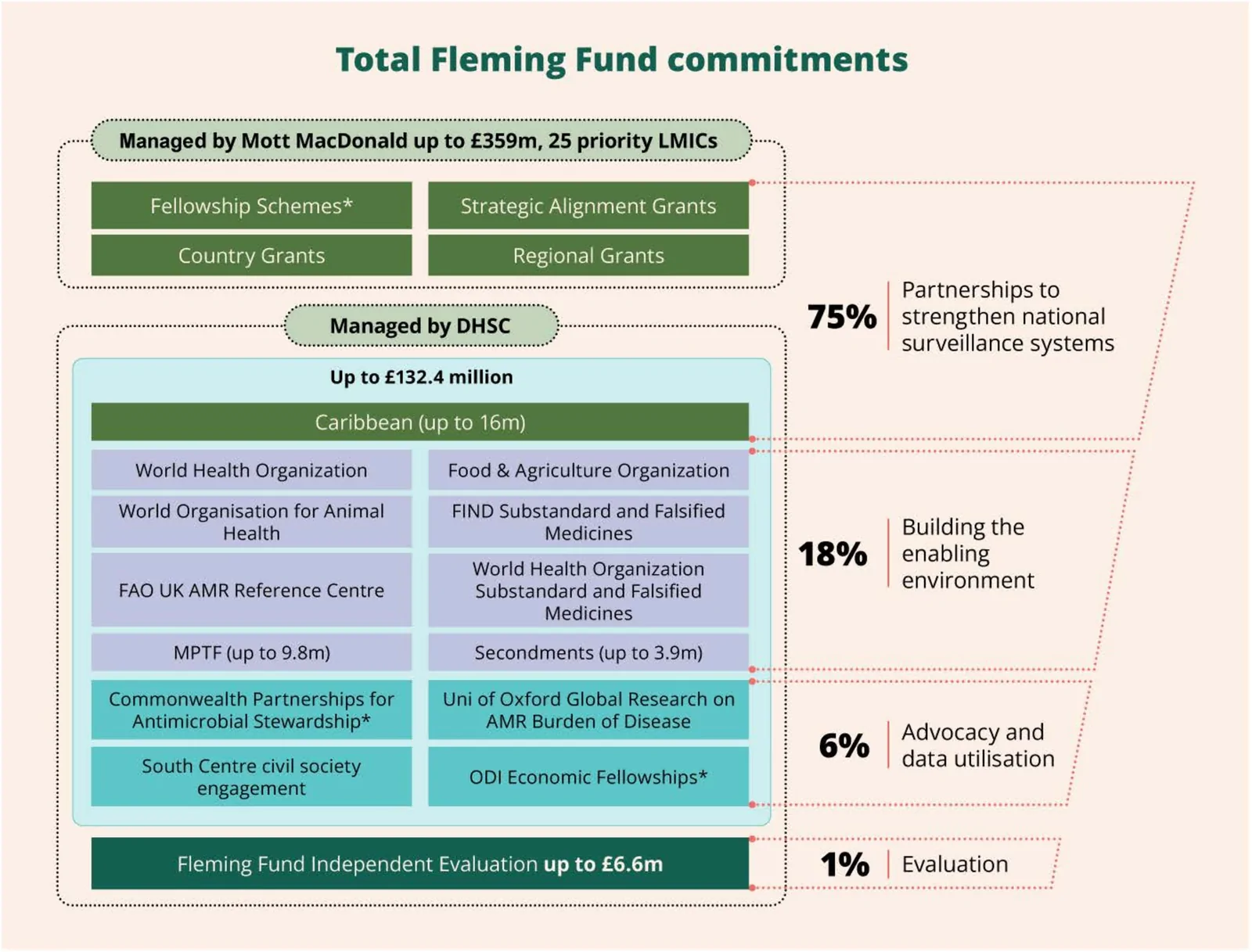
Overview of Fleming fund grant types and financial allocations [25].

An independent evaluation function was embedded within the program from its inception, managed by Itad, reflecting commitment to both accountability, learning and adaptation as essential in complex systems strengthening initiatives.

### Grant Architecture

The work for the Fleming Fund was primarily delivered through a portfolio approach, including more than 60 separate grants across five complementary grant types. Each grant type is summarized below and in Tables 1 and 2, with fuller descriptions in Supplementary Annex II.

- Country grants formed the core of the program and received the majority of the Fleming Fund's investment (Figure 2). These grants supported national surveillance systems through laboratory strengthening, workforce development, governance support, and data systems. Activities were designed to work through national systems and in alignment with national AMR strategies and action plans. Grants were ambitious relative to investment and implementation timeframes (ranging from £2 to 15 million, averaging £7.2 million, over 4–7 years of implementation, covering multiple sectors and surveillance sites).
- Regional grants provided multicountry technical assistance and shared functions that could not be delivered efficiently through individual country grants, including laboratory quality assurance, analytical methods, clinical engagement, economic, and burden-of-disease analysis, and One Health coordination. See Table 1 for more details.
- Strategic Alignment Grants complemented investments by other partners in AMR surveillance and control, responding to opportunities identified during implementation.
- Fellowships supported individual practitioners and leaders working on AMR surveillance and policy, supporting professional development and leadership; Fellows included early- to senior-career government staff (depending on the focus of the Fellowship), and were supported or mentored by a host institution in a technical agency (often a university) outside the Fellows' own country.
- Global and multilateral grants supported global surveillance platforms, analytical initiatives, professional networks, and normative frameworks through WHO [10], the Food and Agriculture Organization (FAO) [11], the World Organization for Animal Health(WOAH) [12], and United Nations Environment Programme (UNEP) [13], South Centre [14], Commonwealth Partnerships for Antimicrobial Stewardship (CwPAMS) [15], FIND [16], and Global Research on Antimicrobial Resistance (GRAM) [17]. See Table 2 for more details.

**Figure 2.**
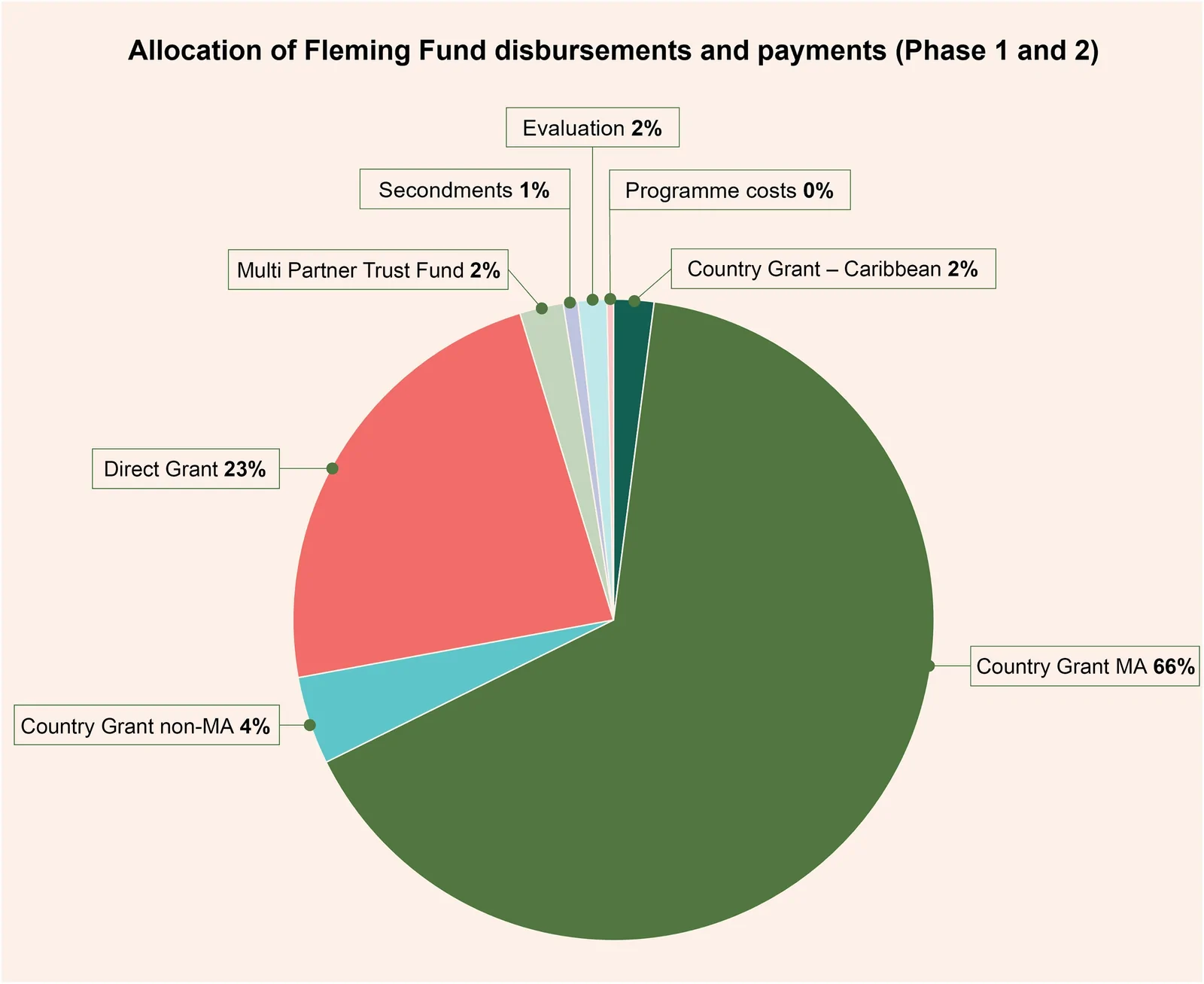
Proportion of Fleming fund disbursements and payments by grant category [25].

**Table 1.** List of Regional Grants (RGs) (Further Detail on Selected Individual Grants Is Provided in Supplementary Annex II)

Timing	Name	Overview
Regional grants—set I (phase 1 and phase 2)	CAPTURA	Strengthens analysis and use of AMR/AMU surveillance data in Asia through retrospective data harmonization, analysis, and capacity building.
	MAAP	Strengthens AMR/AMC data analysis and use in Africa through regional data aggregation, standardization, and capacity building.
	EQuAFRICA	Strengthens external quality assurance (EQA) systems for AMR testing in Africa and supports national laboratory quality frameworks.
	EQASIA	Strengthens EQA systems and professional networks for AMR laboratories in Asia, improving participation, communication, and engagement.
	RADAAR	Strengthens policy uptake of AMR data by addressing bottlenecks, supporting analysis, and developing advocacy guidance.
	QWArS	Strengthens AMR surveillance workforce through training in epidemiology and laboratory microbiology and embedding professional development pathways.
	SeqAfrica	Establishes and expands regional whole-genome sequencing and bioinformatics capacity for AMR surveillance in Africa.
Regional grants—set II (phase 2 only)	Data & Evidence Use (grants in Africa & Asia)	Strengthens use of AMR surveillance data for policy and planning across One Health sectors, including burden and investment analysis.
	AMROH (grants in S Asia, SE Asia, E&S Africa, W Africa)	Strengthens One Health integration of AMR surveillance through improved cross-sectoral coordination and data linkages.
	Gender & Equity	Mainstreams gender and equity in AMR surveillance, capacity building, data use, and policy engagement.
	Clinical Engagement (grants in Africa & Asia)	Strengthens clinical engagement through improved use of laboratory data, antimicrobial stewardship, and diagnostic stewardship.

**Table 2.** Summary of Direct Grants Objectives and Geographic Scope (Further Detail on Selected Individual Grants Is Provided in Supplementary Annex II)

Grantee	Objective	Focus Countries
WHO	Supports implementation and monitoring of AMR NAPs; strengthens AMS and diagnostics; improves data use for policy and programs.	Six to seven countries
FAO	Strengthens data generation, analysis, and use for evidence-based decision making in the food and agriculture sector.	Twelve focus countries
WOAH	Supports development and use of AMR surveillance tools and data in animal health, including antimicrobial use reporting and investment cases.	Combined global reach with targeted country engagement
WHO-SF	Generates evidence on links between substandard/falsified medicines and AMR; supports use of evidence for policy and regulation.	Pilots: Tanzania and Indonesia.
South Centre	Strengthens understanding of cross-sectoral AMR surveillance and evidence-based action.	Global, regional, country.
CwPAMS	Strengthens AMS and IPC structures, knowledge, and practice in LMICs in line with NAPs.	Eight countries
FAO Reference Centre	Builds in-country workforce capacity through research collaboration.	Six countries
MPTF	Strengthens country capacity for One Health AMR responses; supports global and regional coordination and governance.	Fourteen countries
GRAM	Produces global estimates of AMR burden; integrates into GBD; strengthens LMIC data analysis capacity; supports dissemination.	Three LMICs for capacity building, 10 in each WHO region for dissemination.

## THE FLEMING FUND'S APPROACH TO AMR SURVEILLANCE

### Design Logic and Foundations

The Fleming Fund's technical scope was defined around the surveillance pillar of the WHO Global Action Plan on AMR (2015), focusing on the generation, analysis, sharing and use of AMR surveillance data rather than broader AMR control or stewardship interventions. A core objective was to help countries generate data compatible with international systems, particularly GLASS [18], while embedding surveillance within national One Health AMR action plans.

The Fleming Fund's approach was developed during an inception period, informed by DHSC design parameters. A set of program aims and exclusions were set out by DHSC at the start of the Fleming Fund, focused on six key areas:

1. Building laboratory capacity for diagnosis
2. Collecting drug resistance data
3. Enabling the sharing of drug resistance data locally, regionally and internationally
4. Collating and analyzing data on the sale and use of antimicrobial medicines, particularly antibiotics
5. Advocating the application of these data to promote the rational use of antimicrobials for human health, animal health and agriculture
6. Expectations to shape sustainable AMR surveillance systems

DHSC set further parameters to guide design:

1. Country focus: 20 ODA eligible countries in Sub-Saharan Africa, Southern and South Eastern Asia, and with existing UK diplomatic relations
2. Technical focus: detecting and reporting on seven pathogenic bacteria-antibacterial drug combinations identified as of international public health concern; funding a prescribed menu of activities embedded in national public health systems
3. Grant making modality: not allowing financial disbursements directly to governments; subsequently prescribing competitive grant tendering

2015 scoping studies, and the LSHTM roadmap for AMR surveillance LMICs also informed the Fund's design [19]; the roadmap conceptualized AMR surveillance as a staged, system-wide capability requiring coordinated investment across laboratories, workforce, data systems, governance and data use, with sentinel surveillance systems expanding stepwise as capacity developed.

At the time of program design, operational guidance for multisectoral and One Health surveillance was limited, requiring pragmatic design choices and adaptive delivery.

A program-level ToC was developed, after the initial laboratory-focused design, and evolved over time. It articulated (Figure 3) a phased contribution pathway: Phase 1 focused on the foundational components required to generate credible AMR data; Phase 2 on the interpretation and routine use of those data to inform policy, planning and practice, albeit with longer-term outcomes left relatively vague and action toward them necessarily exploratory; and Phase 3 on action on AMR, understood as sustained changes in policy or clinical practice.

**Figure 3.**
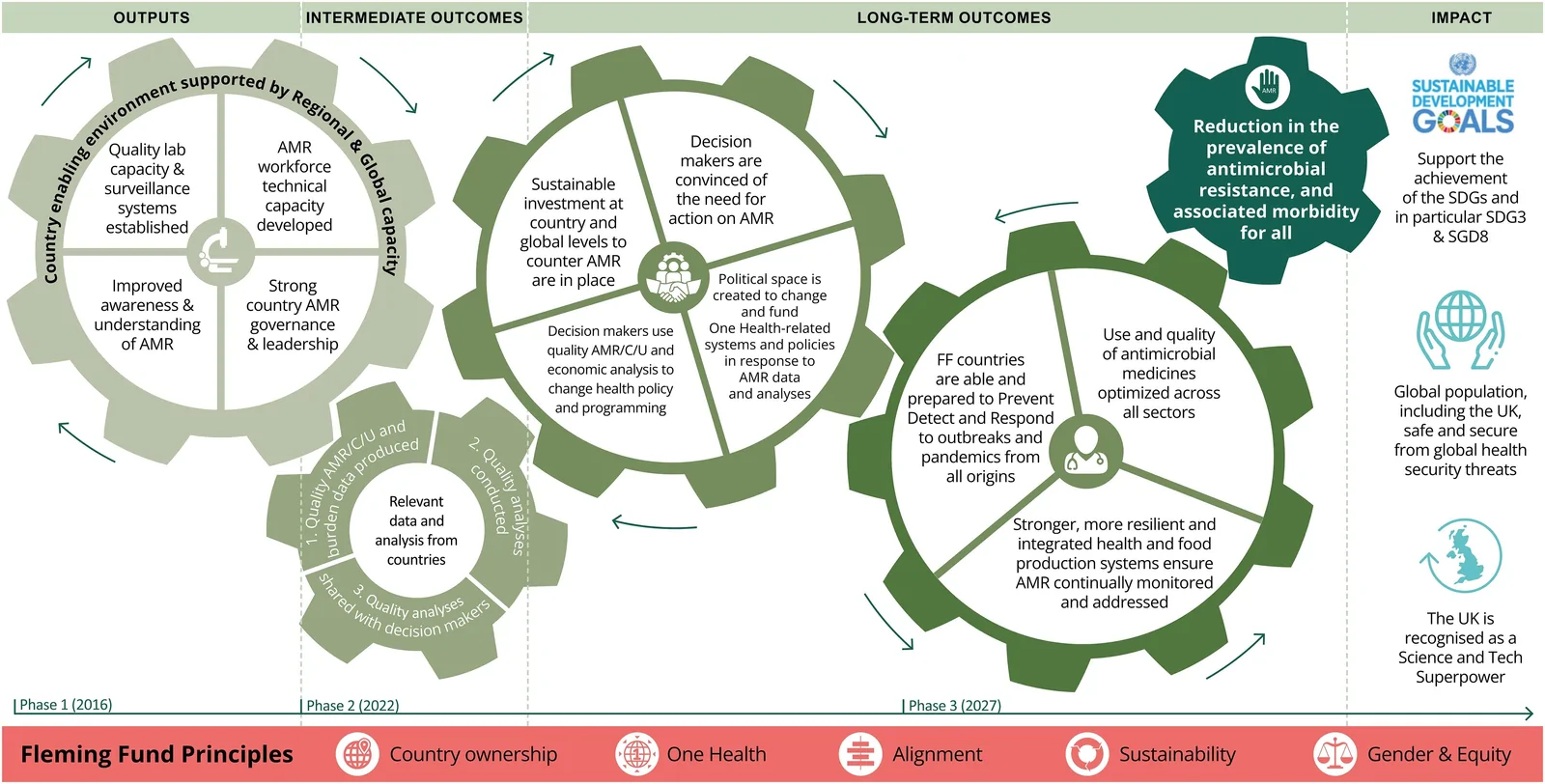
Fleming fund program ToC [25].

Sustainability was central to the Fleming Fund's intent because AMR surveillance is a recurring public function dependent on reliable financing, institutional anchoring, skilled staff, and sustained demand for data. The program's experience highlights the tension between accelerating capacity in the short to medium term and embedding systems that can be maintained and routinely used over the longer-term. The fund incorporated sustainability as an underpinning principle from the outset and considered what could be sustained as support to laboratories was defined; specific sustainability and exit plans were developed by all grantees in Phase 2. Related issues are therefore taken up across the series.

Although conceived as a 10-year investment, the effective period for country-level delivery was closer to seven years once phased start-up, sequencing and replanning are taken into account (Figure 4). This matters for interpreting observed results that depend on institutionalization, routine data use, and sustained financing.

**Figure 4.**
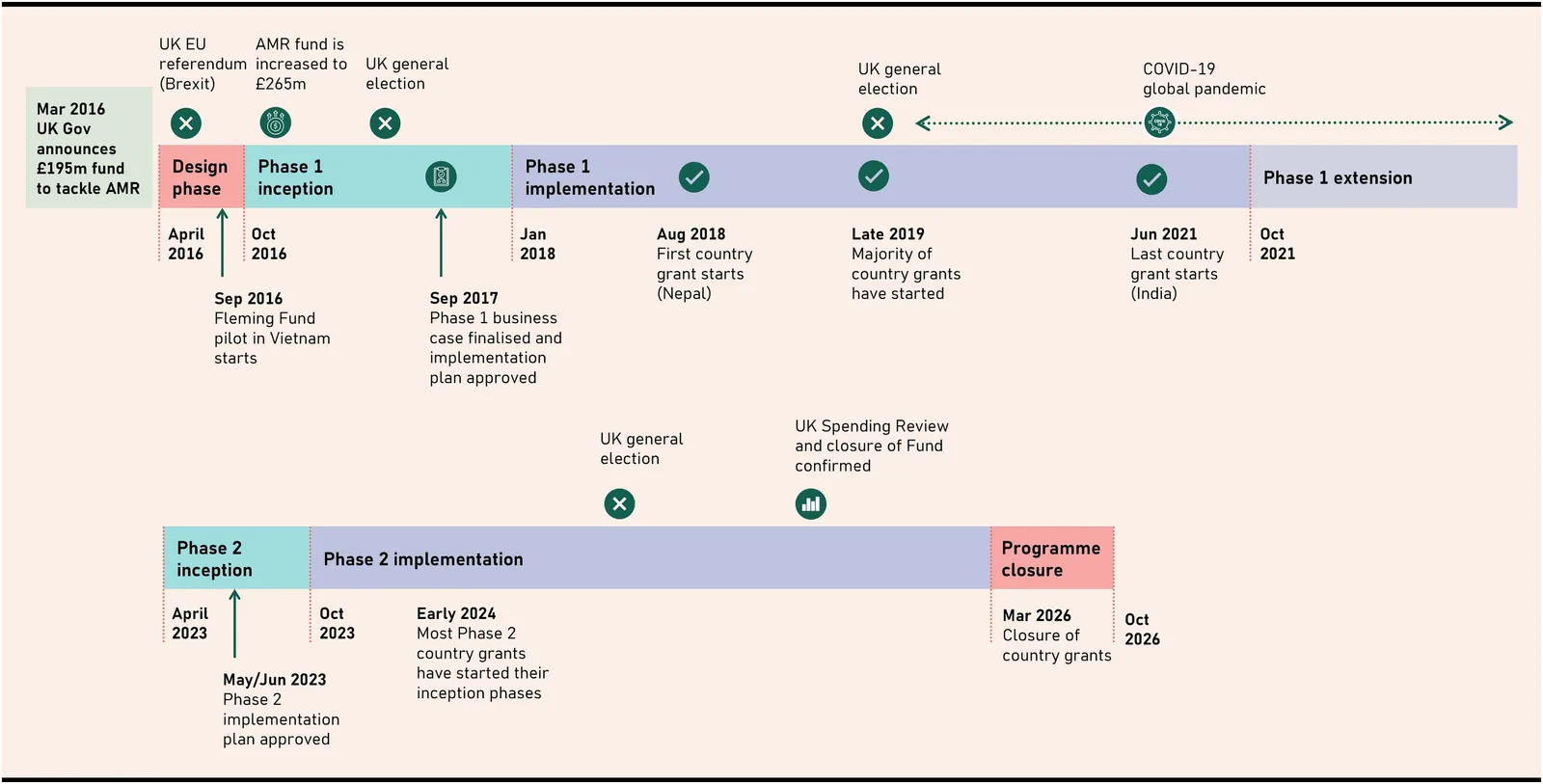
Fleming fund program timeline [25].

### Core System Components for AMR Data Generation (Phase 1 Outputs)

Consistent with the ToC, Phase 1 focused on strengthening interdependent system components required to generate reliable, comparable and interpretable AMR surveillance data, with most investment directed toward laboratory strengthening, supported by workforce development, data systems, governance, and sampling pathways.

#### Laboratory Strengthening for Quality Data Generation

Laboratory strengthening formed the largest component of investment and focused on establishing routine bacteriology and antimicrobial susceptibility testing for priority pathogen–antimicrobial combinations aligned with WHO GLASS and One Health priorities. Interventions typically included laboratory renovation and equipment, standardized testing methods and workflows, strengthened reference laboratory functions, and essential consumables. Quality assurance and standardization were embedded through standard operating procedures, external quality assessment, quality management systems, and accreditation readiness, complemented by regional QA initiatives.

#### Workforce Development

Workforce development supported laboratory scientists, epidemiologists, data analysts and policy actors through in-service training, mentorship, supervision, multidisciplinary surveillance teams, and the Fleming Fellowship program (Box 1).

Box 1. **Fleming Fellows**

The Fleming Fund Fellowship scheme complemented country and regional grants by strengthening human capacity for AMR surveillance. It supported government and public-sector professionals in ministries, national reference laboratories, public health institutes, and related agencies, hosted by technical institutions domestically or internationally. Fellowship profiles spanned laboratory science, epidemiology, data analysis, AMU/C, governance, and policy.

#### Data Systems and Management

Data systems investments supported aggregation, analysis, and reporting at facility and national levels, including implementation or upgrading of laboratory information management systems; use of tools such as WHONET [20] for data analysis; development of national data platforms; and reporting to international systems including GLASS, InFARM [21], and ANIMUSE [22].

#### Sampling Pathways and the Clinical Interface

Because data quality depends on how samples are generated, the Fleming Fund supported improvements in sampling practices and the interface between laboratories and clinicians. This included guidance on appropriate specimen collection with associated clinical and demographic data; strengthening of diagnostic stewardship and clinician–laboratory interaction; and in some contexts (particularly animal health), targeted active sampling to complement routine testing. Across sectors, surveillance prioritized feasible, standardized approaches over statistically representative sampling.

#### Governance and Multisectoral Coordination

Governance and coordination support, in line with AMR National Action Plans, included establishment or strengthening of national AMR coordination committees or equivalent bodies; support to secretariat functions, coordination processes and alignment with National Action Plans; and development of structures for multisectoral engagement and data sharing across human, animal and environmental sectors.

#### Global and Regional Complementary Interventions

Regional and global grants reinforced country-level investments by supporting functions that could not be efficiently delivered at the national level, including quality assurance, proficiency testing, reference laboratory functions, and global surveillance standards and platforms.

### From Data Generation to Use (Phase 2 Strategic Shifts)

During Phase 2, the Fleming Fund placed greater emphasis on interpretation, dissemination and use of AMR data (Box 2), reflecting recognition that data generation alone is insufficient to influence policy or practice (note that the shift on substandard and falsified medicines was the focus of a Fleming Fund grant to WHO, but neither reflected in the ToC (Figure 3) nor part of the independent evaluation scope and so not captured in this series).

Box 2. **Phase 2 Evolution Through Strategic shifts [23]**

Phase II introduced strategic shifts to strengthen the use of AMR data in policy, planning, and clinical practice. Key shifts included:

- **AMR data use:** supporting translation of surveillance outputs into policy, planning, and clinical decision-making.
- **Economic case for AMR investment:** strengthening evidence on disease burden, consumption, and costs of inaction.
- **One Health:** increasing attention to animal health and environmental dimensions of tackling AMR.
- **Substandard and falsified medicines:** generating evidence to inform regulatory and policy responses.

Phase 2 also strengthened attention to facility- and national-level data use, including linking AMR data with complementary evidence and strengthening engagement with clinicians. See Paper 2 for details [5]. Most investment nevertheless remained focused on data generation (around 70% in Phase 2, compared with 80% in Phase 1); with the implications explored in subsequent papers.

### Tailoring Delivery to Country Contexts

Delivery was tailored to country context. From 2018 to 2025, the Fleming Fund supported 25 countries (Figure 5) across four regions, which were grouped using an A–D categorization reflecting the maturity of AMR national action plans and surveillance systems (Bangladesh, Bhutan, Cambodia, Cameroon, Eswatini, Ghana, India, Indonesia, Kenya, Laos, Malawi, Nepal, Nigeria, Pakistan, Papua New Guinea (PNG), Rwanda, Senegal, Sierra Leone, Sri Lanka, Tanzania, Timor-Leste, Uganda, Vietnam, Zambia and Zimbabwe. Note that additional countries, beyond those listed here, were supported through other grant mechanisms such as the MPTF or work in the Caribbean). This informed sequencing, pacing and scale.

**Figure 5.**
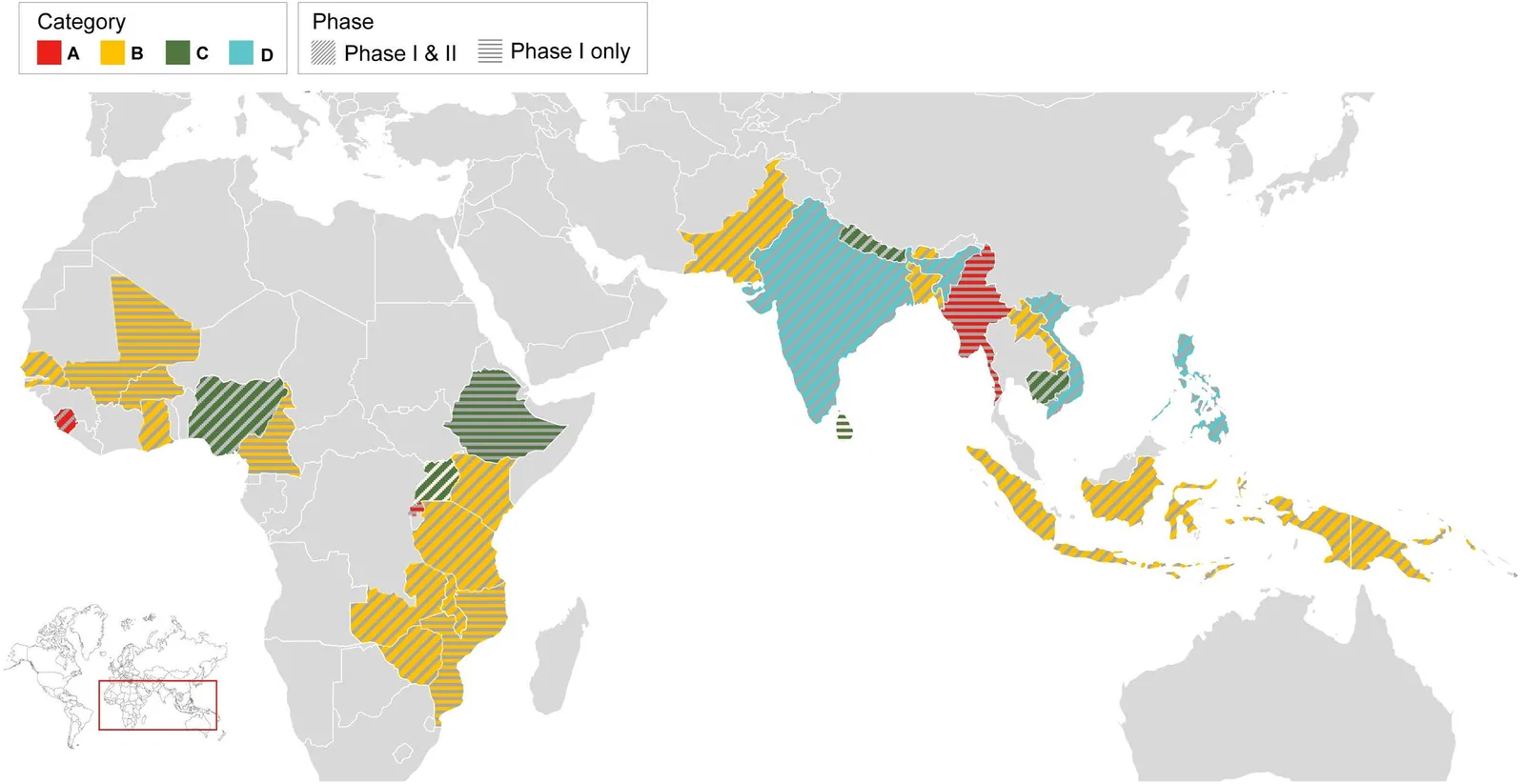
Fleming Fund focus countries. Figure 5 refers to A-D categories which the MA used to assess the strength of each country’s AMR surveillance capabilities at the start of Phase 1 and the start of Phase 2. These categories were described as follows: Category A comprised countries without a National Action Plan (NAP), or where a NAP was at an early or draft stage and not close to finalization; such countries typically lacked functional coordination committees, may not have undertaken situation analyses, and showed limited engagement with the AMR agenda, and were therefore not favored for investment at that time. Category B included countries with a NAP in place but without an operational surveillance system; these countries had often received external support to develop AMR plans and recognized the need for surveillance, but implementation progress was limited largely due to resource constraints rather than an absence of political will, and they were the primary focus of Fleming Fund investments. Category C covered countries where modest surveillance arrangements existed—predominantly in human health—but were small-scale, fragmented and constrained by gaps in quality assurance, analytical capacity, laboratory capability, and sustainability, with weak cross-sectoral integration for One Health. Category D referred to countries with relatively more advanced surveillance systems, in some cases including animal health, where support was expected to be more targeted and additive, aligned with government and other donor investments, and focused on nuanced contributions and strengthening One Health approaches.

In each country, the MA undertook situational analyses of laboratory and surveillance capacity, covering equipment, human resources, data management, quality assurance systems and throughput. These analyses informed priorities, balancing capacity gaps, sustainability, and alignment with national AMR action plans. In many settings, National Action Plans were new and informed primarily by global guidance rather than country-specific surveillance data, so baseline needs were not always well understood.

Laboratory support commonly included infrastructure renovation and equipment provision during Phase 1, including installation of automated blood culture and AST systems in health facility laboratories. In animal health fewer laboratories were supported, reflecting the greater reliance on active surveillance approaches (see other papers in this series for further discussion) [4, 6].

## EMERGING RESULTS

Program monitoring and independent evaluation [24, 25] indicate that the Fleming Fund contributed substantially to strengthening AMR surveillance capacity in diverse LMICs. Reported improvements have been observed in laboratory performance, workforce competence, governance arrangements, and participation in international reporting systems such as GLASS.

Progress was uneven, reflecting differences in starting conditions, political commitment, and external shocks including COVID-19. Nonetheless, the volume of AMR data generated increased across much of the portfolio, alongside improvements in analysis and sharing at national, regional and international levels.

Use of AMR data remained limited and uneven. Where observed, it was clearest at facility level, informing clinical practice, stewardship and infection prevention. At higher system levels, data more often contribute to technical deliberation, priority-setting and periodic policy processes, with less evidence of routine integration into regulatory, financing or system-wide decision-making. This reflects the time required to embed data use and the need for sustained investment in the analytical, governance and decision-making capacities.

The Fleming Fund also helped position AMR surveillance as a routine public health function and has contributed to a global evidence base for stewardship, policy dialogue and international prioritization.

On this basis the Fleming Fund can be understood as a sustained effort to strengthen the foundational systems required for AMR surveillance across diverse contexts, operating at the intersection of national capacity development and global health security. The design choices, implementation experience and constraints described here provide the context for the analyses that follow in this series. The papers that follow examine how these investments have contributed to data generation, data use, multisectoral coordination and future directions for AMR surveillance and action.

## Supplementary Material

ciag499_Supplementary_Data

## Supplementary Data

Supplementary materials are available at *The Journal of Infectious Diseases* online (http://jid.oxfordjournals.org/). Supplementary materials consist of data provided by the author that are published to benefit the reader. The posted materials are not copyedited. The contents of all supplementary data are the sole responsibility of the authors. Questions or messages regarding errors should be addressed to the author.
