# Supplementary material for: The Fleming Fund: Key Features of Design and Approach to Strengthening Antimicrobial Resistance Surveillance in Low- and Middle-Income Countries
Source: Clin Infect Dis. 2026 Oct 19;83(Suppl 1):S3–S12. doi: 10.1093/cid/ciag499 (PMC13679384; doi:10.1093/cid/ciag499)
Supplement: Supplementary file 1 [file ciag499_supplementary_data.docx]

**Annex**

**Annex I. Supplementary Methods: Approach to Evidence and Synthesis Across the Series**

**Purpose and positioning of the series**

This series synthesises learning from the Fleming Fund, drawing on programme implementation experience, independent evaluation evidence and practitioner perspectives. It aims to inform future policy and practice on AMR surveillance system development and use in LMICs.

The approach is a narrative, learning-oriented synthesis rather than a formal systematic review or independent evaluation. It draws on evaluative material but does not seek to establish causal attribution or assess programme performance against predefined criteria. Instead, it identifies cross-cutting patterns, lessons and insights from implementation experience

across diverse contexts.

**Overall methodological approach**

The series used a structured participatory synthesis process combining targeted evidence review with iterative multi-author engagement. Each paper was produced by a writing group convened to contribute to scoping, drafting and review.

Across the series, papers followed a consistent process of evidence identification, iterative drafting, structured discussion and consolidation of findings, through claims grounded in identifiable evidence sources wherever possible.

**Evidence base**

The series draws on programme documentation, evaluation outputs, technical reports, surveillance data, international reporting and relevant academic and grey literature. AMR data generated were country-owned and only available for the MA or evaluation team where shared publicly.

Evidence selection was purposeful rather than comprehensive, prioritising material relevant to each paper’s scope. Sources were prioritised where they provided insight into implementation experience, system functionality and reported outputs. Claims and examples were included where supported by identifiable sources.

**Participation, authorship and expertise**

Author groups were convened through a structured invitation process and included multidisciplinary contributors from across the Fleming Fund network. This process was led by a lead or coordinating author from the Itad^[1]^ evaluation team, with support from an Itad ‘secretariat’ function.

Groups were designed to draw on direct implementation experience from individuals involved in delivering, managing, supporting or evaluating AMR surveillance systems. Contributors included AMR focal points, laboratory specialists, clinicians, policy-makers, programme implementers and evaluators across human, animal and environmental health sectors, from countries in Africa and Asia as well as regional and global experts. This provided practical, day-to-day insight into how programme implementation worked, including strengths, weaknesses, trade-offs and adaptations. Contributions informed interpretation and conclusions through iterative discussion and review.

**Process for synthesis and development of papers**

Each paper followed a consistent process of scoping, evidence collation, iterative drafting, structured discussion, review and consolidation. Contributors proposed examples, challenged interpretations and refined findings; claims were retained where supported by identifiable evidence.

**Analytical framing**

The Fleming Fund programme theory of change provided a common lens for interpreting how investments in systems and capacity to improved data generation and use.

**Limitations**

The series combined UK-based coordination with substantive input from country-level experts to strengthen relevance and grounding in implementation realities. This introduces risks of selection and confirmation bias, as many contributors were directly involved in programme implementation.

The evidence base was selected for relevance to each paper’s scope and was not intended to be comprehensive. In addition, much of the AMR data generated through the programme remained under country ownership and was available to the MA or evaluation team only where shared publicly. Findings should be interpreted as plausible lessons rather than definitive conclusions. Unsuccessful interventions or negative results may be under-represented. Constraints, partial implementation and areas of limited progress was considered, although decisions about inclusion and emphasis were ultimately made through discussion and consensus within the drafting groups.

**Conflict of interest**

No reported conflicts of interest. All authors have submitted the ICMJE Form for Disclosure of Potential Conflicts of Interest. Conflicts that the editors consider relevant to the content of the manuscript have been disclosed.

Authors include individuals involved in the design, implementation, technical support and evaluation of the Fleming Fund. The series should therefore be understood as a programme-linked synthesis rather than an independent assessment.

**Annex II. Selected Regional and Direct Grants – Extended Descriptions**

This annex provides extended descriptions of selected grants summarised in the main text.

| **Grant** | **Extended description** |
| --- | --- |
| CAPTURA | Provides regional technical assistance in Asia to strengthen analysis and use of AMR and AMU surveillance data, including retrospective data collection, harmonisation, and capacity building for evidence-informed policy and clinical decision-making. |
| MAAP | Supports AMR and antimicrobial consumption data use in Africa through African-led aggregation, standardisation, and analysis of large-scale laboratory and consumption datasets, alongside capacity building for regional and national evidence generation. |
| RADAAR | Strengthens the policy relevance and uptake of AMR surveillance data by identifying bottlenecks, developing advocacy guidance, and supporting improved analysis, sharing, and use of data in national and regional policy processes. |
| Data & Evidence Use (Africa and Asia) | Provides regional technical assistance to strengthen use of AMR surveillance data for policy, regulation, and planning across One Health sectors, including support for burden estimation and development of investment cases aligned with country strategies. |
| AMROH (all regions) | Supports Phase II strategic shifts by strengthening One Health integration of AMR surveillance across human and animal health systems, focusing on cross-sectoral coordination, data linkages, and alignment with country investment strategies. |
| Gender & Equity | Mainstreams gender and equity across the programme through landscape analyses and integration of gender- and equity-responsive approaches into surveillance, capacity building, data use, and policy engagement at country and regional levels. |
| Clinical Engagement (Africa and Asia) | Strengthens clinical engagement in AMR surveillance and data use, including improved diagnostic and antimicrobial stewardship, enhanced use of laboratory services, and stronger linkages between clinicians and laboratories. |
| GRAM | Produces global and country-level estimates of AMR burden by pathogen, drug combination, age, sex, and location; integrates these into the Global Burden of Disease framework; strengthens LMIC capacity for AMR data analysis; and supports dissemination of findings for policy and research use. |

1. Itad ([www.itad.com](http://www.itad.com)) was the independent evaluation and learning partner for the Fleming Fund from 2016 to 2026. DHSC asked Itad to coordinate and facilitation the production of this series as an extension to this role.
